# Polynomial force approximations and multifrequency atomic force microscopy

**DOI:** 10.3762/bjnano.4.41

**Published:** 2013-06-10

**Authors:** Daniel Platz, Daniel Forchheimer, Erik A Tholén, David B Haviland

**Affiliations:** 1 KTH Royal Institute of Technology, Section for Nanostructure Physics, Albanova University Center, SE-106 91 Stockholm, Sweden; 2 Intermodulation Products AB, Vasavägen 29, SE-169 58 Solna, Sweden

**Keywords:** AFM, atomic force microscopy, force spectroscopy, multifrequency, intermodulation, polynomial

## Abstract

We present polynomial force reconstruction from experimental intermodulation atomic force microscopy (ImAFM) data. We study the tip–surface force during a slow surface approach and compare the results with amplitude-dependence force spectroscopy (ADFS). Based on polynomial force reconstruction we generate high-resolution surface-property maps of polymer blend samples. The polynomial method is described as a special example of a more general approximative force reconstruction, where the aim is to determine model parameters that best approximate the measured force spectrum. This approximative approach is not limited to spectral data, and we demonstrate how it can be adapted to a force quadrature picture.

## Introduction

The combination of high-resolution imaging [1–4] and high-accuracy force measurements [5–10] is a strong driving force for the development of atomic force microscopy (AFM). The advent of multifrequency AFM resulted in a variety of new measurement techniques enabling enhanced contrast and spatial mapping of surface properties on a wide range of samples [11]. However, multifrequency AFM creates more data than conventional AFM, which both complicates the interpretation of measurement results and offers the possibility of much more detailed surface analysis. One of the goals when interpreting AFM data is the reconstruction of the force between a surface and the sharp tip at the end of the oscillating cantilever, while scanning. This reconstruction is readily possible by means of the Fourier transform if the motion of the tip in response to this force and the linear response function of the cantilever are known over a wide frequency band [12–15]. However, the tip motion is often only measurable in a narrow frequency band around a cantilever resonance, since the cantilever transfer function sharply attenuates other frequency components of the tip motion, placing them below the detection noise floor. With this measured partial motion spectrum, the original force cannot be recovered with a simple Fourier transform, and additional assumptions about the functional representation of the tip–surface force are required.

These assumptions, which can be expressed with a finite set of parameters, result in a correlation of the measurable and the nonmeasurable frequency components of the motion. The parameters are chosen such that the spectrum of the reconstructed force best approximates the measured partial force spectrum. This approximative reconstruction requires the use of numerical optimization techniques if the force model is nonlinear in the parameters [16]. Analytic solutions can be obtained if the model is linear in the parameters [9,17–19]. Such a linear model of particular interest is the polynomial, as it constitutes a general expansion of the tip–surface force.

Polynomial force reconstruction methods have been proposed theoretically and tested on simulated data for intermodulation AFM (ImAFM) [17,19]. Here, we demonstrate, for the first time, polynomial force reconstruction on experimental ImAFM data and compare it with reconstruction based on amplitude-dependence force spectroscopy (ADFS) [20]. Moreover, fitting a force model to the polynomial reconstruction allows for the extraction of properties such as surface adhesion, sample stiffness or interaction geometry. We demonstrate this extraction of surface properties with high-resolution stiffness maps on a blend of polystyrene (PS) and poly(methyl methacrylate) (PMMA).

Polynomial reconstruction, and most other multifrequency methods, work directly on the measured spectral data of the tip motion. Since the tip motion can be very complicated, the interpretation of spectral data often becomes rather difficult and alternative data-representation schemes might provide a better understanding of the tip–surface force. Recently, we have shown how a narrow-band ImAFM measurement yields the oscillation-amplitude dependence of a force component *F_I_* in-phase with the sinusoidal tip motion and a force component *F_Q_* quadrature, or 90 degrees phase-shifted, to the tip motion [21]. Here we show how polynomial force reconstruction can be performed within the context of this picture of two force quadratures.

## Results and Discussion

### Polynomial force reconstruction from spectral data

In narrow-band AFM the tip dynamics as a function of time *z*(*t*) is usually described by a harmonic oscillator [22–23], subject to an external drive force and a time-dependent tip–surface force

[1]
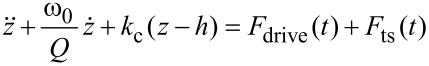


where the dot denotes differentiation with respect to time, ω_0_, *Q* and *k*_c_ are the mode’s resonance frequency, quality factor and spring constant respectively, and *h* is the static equilibrium position of the tip above the surface. One should note that the time dependence of the tip–surface force *F*_ts_ can be considered as an implicit time dependence, since it is assumed that the tip–surface interaction depends on the instantaneous tip position *z* and velocity 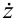, which are functions of time. In Fourier space Equation 1 becomes

[2]
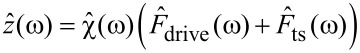


where the linear response function

[3]
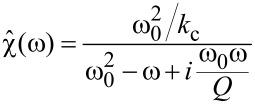


with the complex unit *i* determines the tip response to a sinusoidal force applied at the frequency ω. The drive force can be readily determined from a measurement of the tip motion far away from the surface, *z*_free_(*t*), where the tip–surface force is zero,

[4]
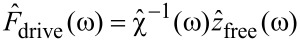


If the broad-band tip response close to the surface 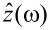 is known, one can easily solve Equation 2 for the spectrum of the tip–surface force

[5]
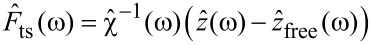


With the inverse Fourier transform, the time-dependent force acting on the tip can be readily determined from Equation 5.

Since the result of an experiment is a vector 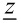 of time-discrete samples of the continuous signal *z*(*t*) during a time window of length *T* = 1/Δω, the Fourier transform can be expressed using a unitary matrix 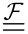,

[6]
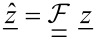


[7]
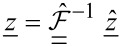


where a single underline denotes a vector and a double underline a matrix. In a real experiment, only a partial motion spectrum 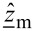 can be measured since the linear transfer function of the cantilever 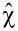 suppresses the response far away from resonance. Mathematically, this can be expressed with a diagonal windowing matrix operator 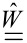 that sets all frequency components outside the resonant detection band to zero. The measured spectrum is then given by

[8]
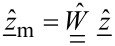


and Equation 5 for the measured data becomes

[9]
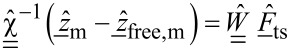


Since 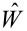 is not invertable we cannot determine the complete force spectrum 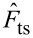 from Equation 9, and thus the time-dependent force remains unknown. To reconstruct the complete force spectrum from the measured partial motion spectrum 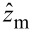, we expand the tip–surface force into a finite series from a set of basis functions, 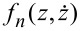 with constant coefficients *g*_n_

[10]
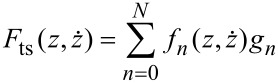


Our assumption that the force can be expanded in this manner results in a correlation of the unknown frequency components of the motion with the measurable components. A common choice for the functions *f_n_* to model conservative forces are monomials [17]

[11]
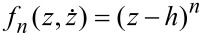


but also other basis functions of the form

[12]
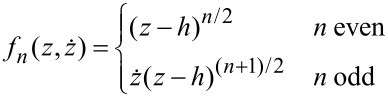


for the representation of position-dependent viscosites have been considered [19]. For a measured tip motion the force vector 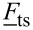 can the be approximated as

[13]
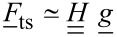


where the coupling matrix *H* is given by

[14]
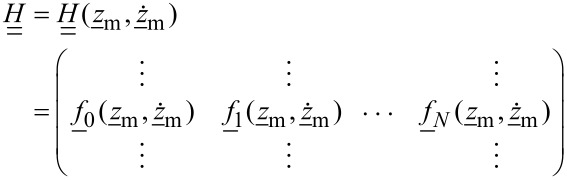


in which the columns are formed by the vectors 
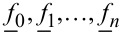
 evaluated at the measured discrete tip positions and velocities. Here, we assume that the measured, or windowed tip motion 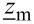 is a good approximation of the complete tip motion 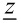. In Fourier space, Equation 13 becomes

[15]
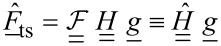


where

[16]
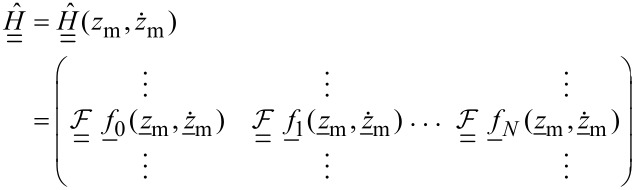


The force matrix Equation 9 can then be written as

[17]
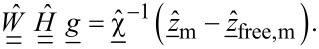


We introduce

[18]
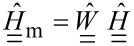


and solve Equation 17 for 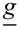 such that

[19]
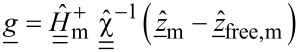


where 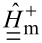 denotes the pseudo-inverse of 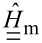. If a unique solution for the coefficient vector 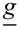 exists, the matrix 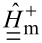 equals the exact inverse of 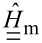. If there is more than one solution for 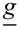, Equation 19 computes the solution for which the vector 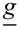 has minimum length. If no solution to Equation 17 exists the pseudo-inverse 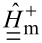 approximates the inverse in a least-squares sense.

The matrix 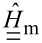 can be rapidly computed from Equation 16 using the Fast Fourier Transform (FFT) algorithm. Therefore, Equation 19 provides an efficient way to determine the expansion coefficients 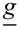 of the the tip–surface force. However, special care should be taken to avoid aliasing effects due to the finite sampling of the data. To increase the numerical stability of 19 it is advantageous to normalize 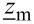 and 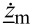 such that the largest absolute value of any vector element is 1. This normalization can be interpreted as a preconditioning procedure for the matrix 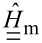.

To further investigate what information about the tip–surface force can be extracted, we focus on the monomial expansion basis defined in Equation 11 and the case of narrow-band ImAFM where the windowing matrix is given by

**Figure 1:**
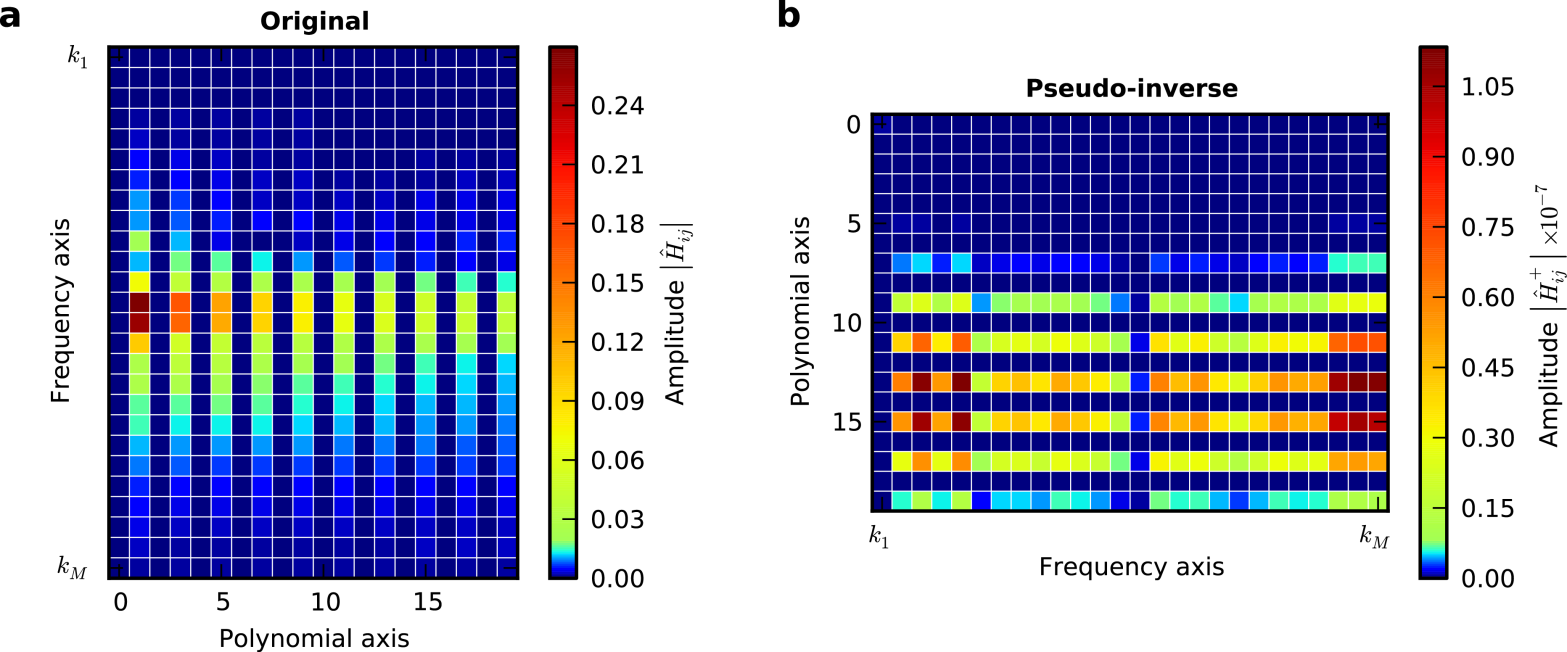
Visualization of the 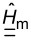 matrix (a) and its pseudo-inverse (b). Only rows with non-zero elements are displayed.

[20]
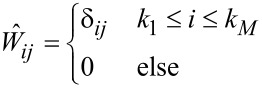


with δ_ij_ being the Kronecker delta, *k*_1_Δω the lower frequency limit of the resonant detection band and *k_M_*Δω the upper limit. In Figure 1 we plot the absolute values of the components of the matrix 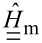 for experimental data. One could imagine applying different windowing matrices when building 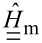, for example one which is weighted by the signal-to-noise ratio at each frequency.

The absolute value of the element of 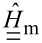 with the index (*i*, *j*) is a measure of how much the *j*-th element *g_j_* of the expansion coefficient vector 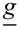, contributes to the force at the *i*-th frequency *i*Δω in the force spectrum 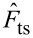. It is apparent from Figure 1 that only polynomial coefficients of odd order contribute to the force measured in the resonant detection band when two drives close to resonance are used in ImAFM. Thus, Equation 19 only yields the odd coefficients in the polynomial force expansion and the resulting polynomial force is odd with respect to *z* = *h*. To determine the missing even coefficients we assume that the tip–surface force is zero for *z* ≥ *z*_non–interacting_. With this assumption we fit the even polynomial coefficients while keeping the odd coefficients constant. This reconstruction method has been extensively tested and its accuracy verified with simulated data [19]. In the following we will show results for experimental data.

### Polynomial force reconstruction during slow surface approach

To demonstrate the capabilities of the polynomial force reconstruction we perform an ImAFM approach measurement on a silicon oxide surface. In this measurement two drive frequencies close to resonance result in a beat-like tip motion, with rapid sinusoidal oscillations and a slowly varying amplitude. The AFM z-piezo moves slowly towards the surface, such that during one beat period the static tip height above the surface can be considered to be constant.

**Figure 2:**
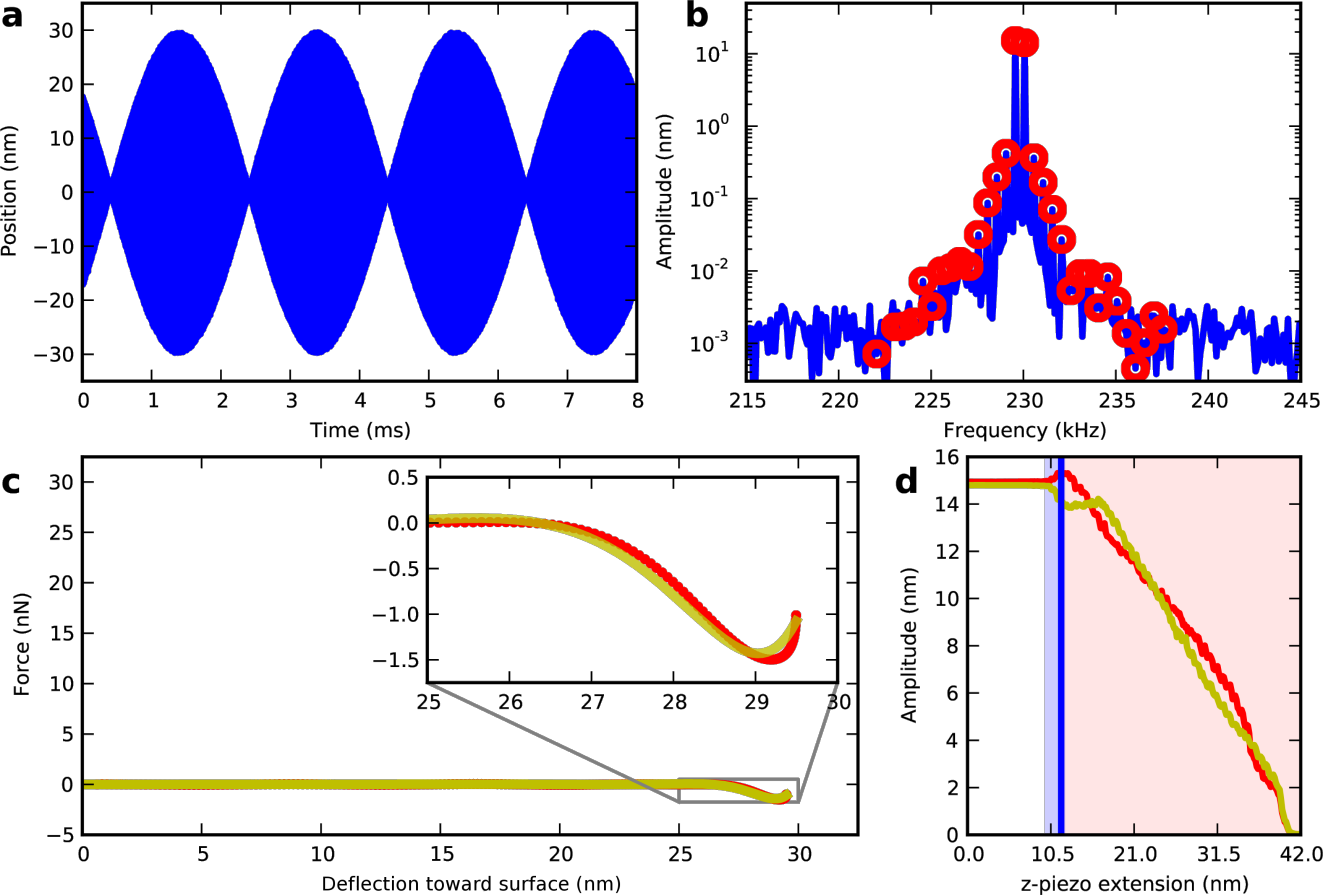
One frame from a surface-approach movie (see Supporting Information File 1) showing the very onset of repulsive forces. The beating waveform (a) has the intermodulation spectrum around resonance (b) where the red circles are analyzed to reconstruct the tip–surface force (c) using both polynomial (yellow) and ADFS (red) methods. The z-piezo extension *z*_piezo_ = 11.8 nm is indicated by the blue vertical line in (d) which displays the amplitudes at the two drive frequencies. The interaction is purely attractive in the blue shaded area, becoming repulsive in the red shaded area.

Figure 2 shows one frame from a movie (Supporting Information File 1) visualizing the measurement. For four consecutive beats in the time domain the corresponding amplitude spectrum around the first resonance is displayed in Figure 2b where the components or partial spectrum used for force reconstruction are marked with red circles. The polynomial force reconstruction is plotted (yellow solid line) in Figure 2c together with an ADFS reconstruction using the same data (red circles). In Figure 2d the amplitudes of the tip motion at the lower (red) and the higher drive frequency (yellow) are shown as functions of the z-piezo extension, and the vertical blue line indicates the current z-piezo extension.

Far away from the surface the tip does not experience any surface force and the motion spectrum exhibits response only at the driven frequencies (Supporting Information File 1). Consequently, the reconstructed force is zero. As the surface is approached, the attractive force regime due to the van der Waals forces between the tip and the surface is reached. In this regime new frequency components appear in the motion spectrum, so-called intermodulation products. Note that in the time domain, the distortion of the signal is barely visible. Both polynomial and ADFS reconstruction show a growing attractive interaction until a force minimum of −1.75 nN is reached at a piezo extension of 11.8 nm. At this point the tip experiences hard mechanical impacts on the sample surface near the beat maximum, which are manifest in the sharp onset of repulsive force in the polynomial and ADFS reconstruction Figure 2c.

**Figure 3:**
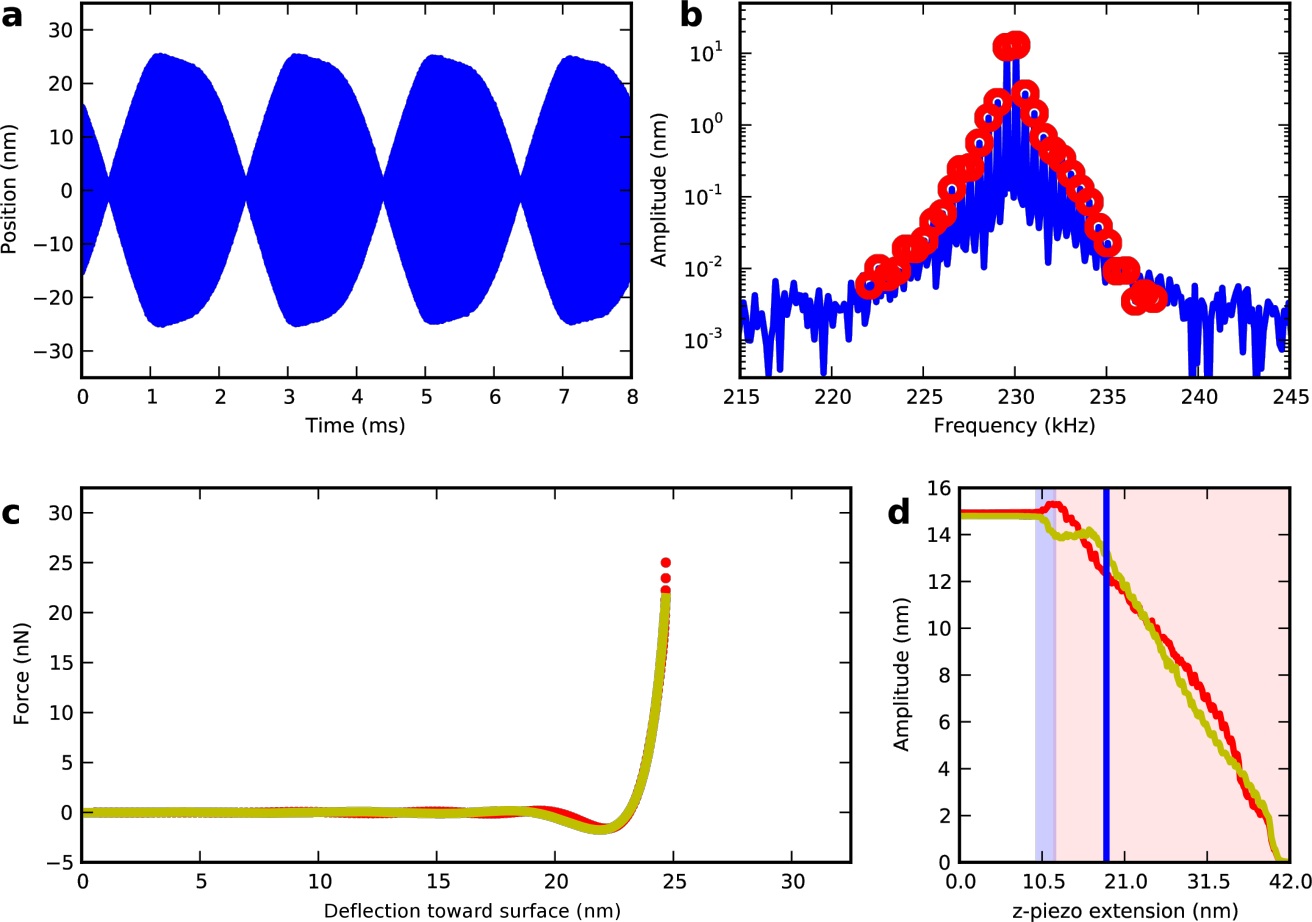
One frame from a surface approach movie (see Supporting Information File 1) showing strongly repulsive forces. The beating waveform (a) has the intermodulation spectrum around resonance (b) where red circles are analyzed to reconstruct the tip–surface force (c) using both polynomial (yellow) and ADFS (red) methods. The z-piezo extension *z*_piezo_ = 18.7 nm is indicated by the blue vertical line in (d), which displays the amplitudes at the two drive frequencies. The interaction is purely attractive in the blue shaded area, becoming repulsive in the red shaded area.

As the z-piezo further extends the tip indents deeper into the surface and experiences stronger repulsive forces as shown in Figure 3 where one frame of the movie at a piezo extension of 18.7 nm is shown.

In this repulsive regime the polynomial and the ADFS reconstruction agree very well. However, the force minimum has a slightly sharper shape with the ADFS reconstruction, which is not constrained to be continuous in high-order derivatives, as for a polynomial. The repulsive force reaches its maximum of 31 nN at a z-piezo extension of 23.6 nm. Moving closer to the surface the maximum force during one beat decreases until the oscillation vanishes.

During the whole surface approach the polynomial and the ADFS force reconstruction agree very well, indicating that the polynomial reconstruction accurately reproduces the force. The shape of the reconstructed force is very stable during the entire approach for both polynomial and ADFS reconstruction, which is a result of the high signal-to-noise ratio for the measured frequency components close to resonance. The stability of the reconstruction during approach gives us confidence in the method’s ability to accurately reconstruct sharp features in the force curve, such as the force minimum.

### Surface parameter mapping

Material scientists are often interested in determining surface properties with high spatial resolution. ImAFM measurements can be performed while scanning a sample surface [24–25], enabling polynomial force reconstruction at every point of an AFM image. A specific force model can then be fitted to the complete force curve or parts thereof, generating a map of the model parameters over the complete surface. To demonstrate this we scanned the surface of a PS/PMMA blend with ImAFM. To the repulsive part of the polynomial force reconstruction we fit a Derjaguin–Muller–Toropov (DMT) force model [26] of the form 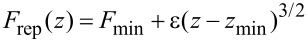 where *z*_min_ is the position of the force minimum *F*_min_ and ε is the DMT stiffness factor, which depends on the tip radius and the effective stiffness of the tip–surface system. One should be aware of the fact that macroscopic force models, such as the DMT model, might not be applicable on the nanoscale [27] and that tip shape and surface topography lead to an interaction geometry that is different from the model geometry of a perfect sphere and a perfectly flat plane [20]. Moreover, the DMT model does not account for adhesive forces in the contact regime, which we try to circumvent by using tips with small radii. While the DMT model provides sufficient insight into material properties, the extracted numerical values of the DMT parameters should not be expected to agree with values for the bulk material.

**Figure 4:**
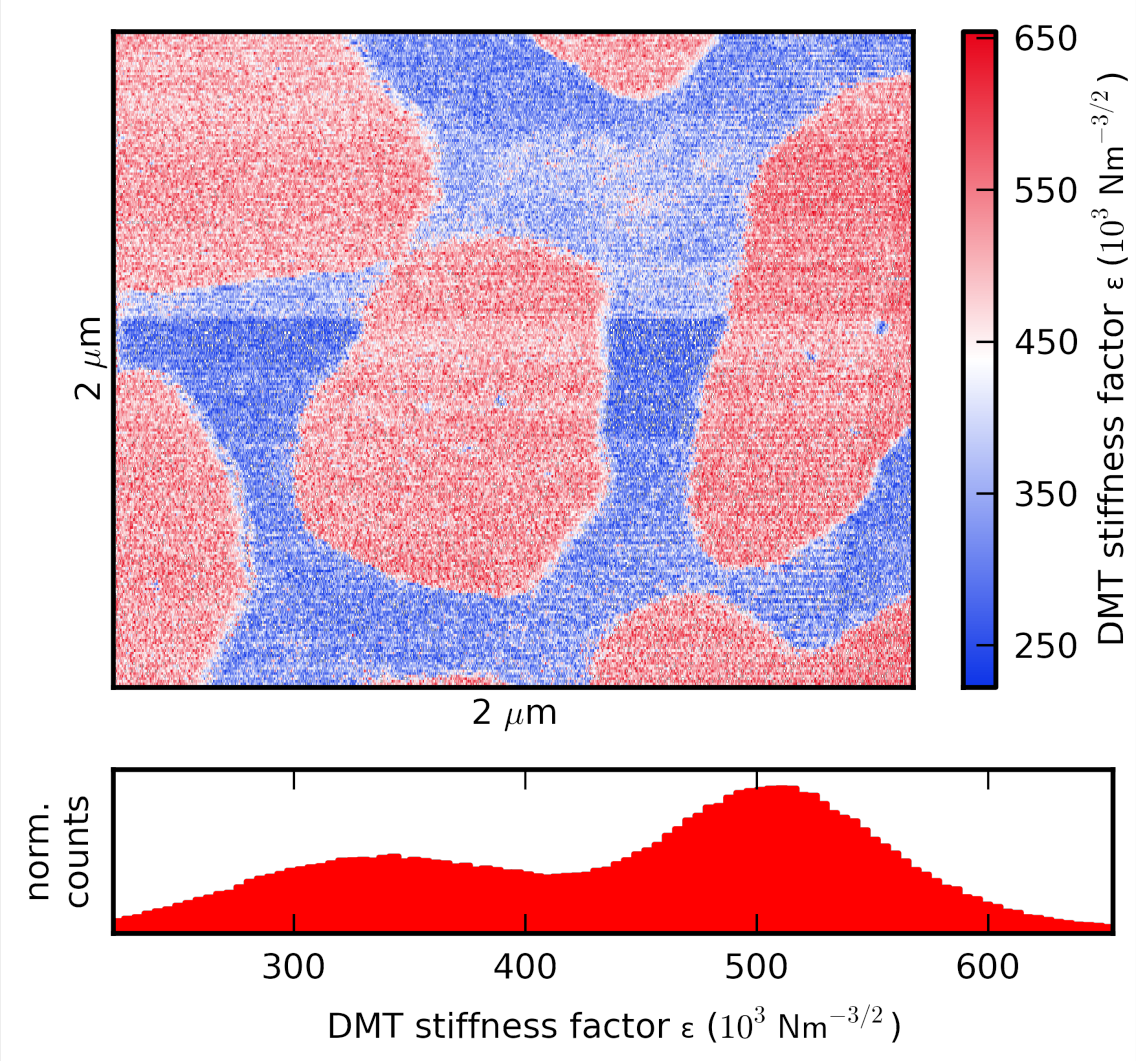
A surface property map showing the DMT stiffness factor ε [N m^–3/2^] with a histogram of the stiffness factor.

In Figure 4 a map of the DMT stiffness factor is shown. Even though the two polymers are very similar in stiffness at room temperature [28], two domains of different stiffness are clearly visible in the stiffness-factor map. The stiffer domains are PMMA-rich and 10 nm higher than the surrounding matrix, which is PS-rich and is a factor of two softer than the PMMA-domains. Similar results on the same model polymer system have been obtained with methods employing higher harmonics [15,29] and by ADFS [20].

### Polynomial reconstruction from force quadrature data

Polynomial force reconstruction is an approximative reconstruction method applied to spectral data obtained from Fourier analysis of the tip motion. The general idea, to determine the parameters of a force model such that an experimental observable is best approximated, is not limited to spectral data. An alternative to the spectral representation is a picture that represents the data in terms of two force quadratures. The force quadrature *F_I_* is the component of the (time-dependent) tip–surface force that is in phase with the sinusoidal tip motion, the quadrature *F_Q_* is the force component that is phase-shifted 90 degrees to the tip motion [21].

[21]
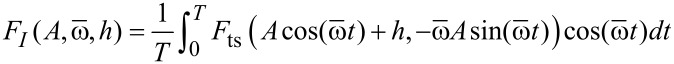


[22]
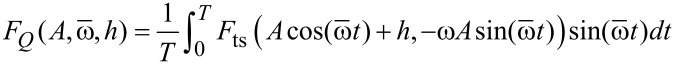


where *T* is the measurement time. The force quadratures are functions of the oscillation amplitude *A*, the oscillation frequency 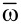 and the static tip height *h*, all of which are constant during each oscillation cycle. However, here we consider only the amplitude dependence of *F_I_* and *F_Q_*, which can be rapidly measured with ImAFM using a single-oscillation-cycle analysis that is based on a separation of time scales [21].

The representation of the measurement result in terms of the force quadratures *F_I_* and *F_Q_* has the advantage that they are directly connected to the tip–surface force and independent of the actual complicated multifrequency tip motion. With spectral data, certain points on the tip–surface force curve will receive greater weight if the tip spends more time at these positions. On the *F_I_*(*A*) and *F_Q_*(*A*) curves the weight at each amplitude can be controlled by design. Furthermore, distortions due to feedback artifacts can easily be removed from the *F_I_*(*A*) and *F_Q_*(*A*) curves, and both conservative and dissipative forces can be analyzed separately.

To demonstrate approximative force reconstruction on force quadrature data, we consider again a conservative polynomial force representation as in Equation 11. For such a force and *h* = 0, *F_Q_*(*A*) = 0 and Equation 21 becomes

[23]
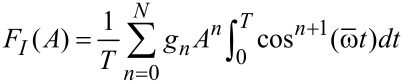


The integral is nonzero only for odd *n* and by using

[24]
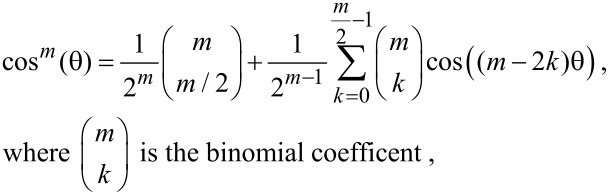


Equation 23 becomes

[25]
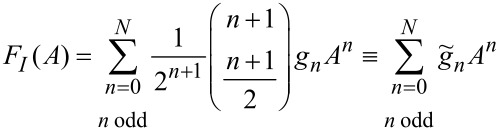


which implies that the odd polynomial coefficients of the force expansion can be obtained by simple rescaling of the coefficients of a polynomial approximation of the *F_I_*(*A*) curve,

[26]
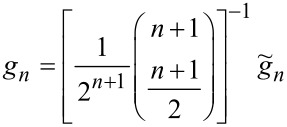


To obtain the even coefficients we apply the same algorithm as for reconstruction from spectral data.

We obtain a polynomial approximation of *F_I_*(*A*) by a simple polynomial fit to equidistant discrete points on the *F_I_*(*A*) curve. Alternative methods such as expansion in orthogonal polynomials or different types of interpolation polynomials with different convergence properties can also be applied. The polynomial reconstruction based on force quadrature data can be implemented even more efficiently than the reconstruction on spectral data since multiple Fourier transforms to construct the coupling matrix 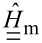 in Equation 16 are not required.

**Figure 5:**
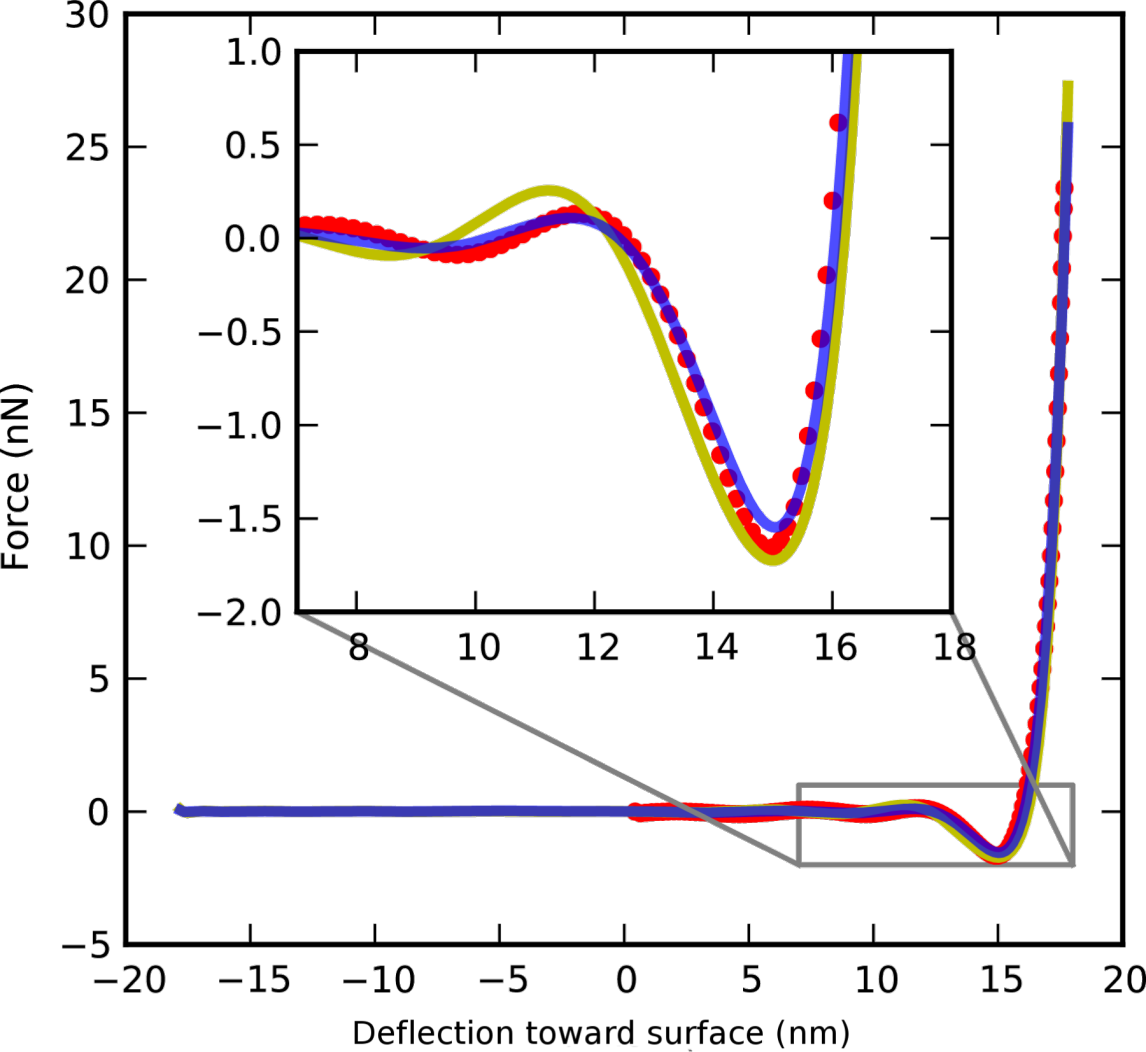
Comparison of force reconstructions for a tip interacting with silicon oxide. Polynomial reconstruction from spectral data (yellow), force quadrature data (blue) and ADFS reconstruction (red circles) are in good agreement.

To validate the equivalence of polynomial force reconstruction on spectral and force quadrature data, we consider the ImAFM approach measurement on silicon oxide described above. From the tip motion at a z-piezo extension of 25.6 nm we compute the *F_I_*(*A*) curve and remove all data points for which the oscillation amplitude was decreasing. From the polynomial approximation of the *F_I_*(*A*) curve we obtain the force polynomial as described above. The resulting reconstruction is shown in Figure 5 (blue line) together with the polynomial reconstruction from spectral data (yellow line) and an ADFS reconstruction (red circles). Over the full range of oscillation both curves agree well. Good agreement is also observed at all other z-piezo extensions.

## Conclusion

Multifrequency AFM opens the window to a wide variety of novel AFM measurement techniques enabling a much improved understanding of the forces between the tip and the surface. We showed that polynomial force reconstruction is an intuitive and powerful method to approximate this interaction, and we demonstrated the method’s use for accurate and detailed force measurement and for high-resolution surface-parameter mapping with experimental data.

As the field of multifrequency AFM continues to evolve, new alternative data-representation schemes can help to simplify analysis and extract more specific properties of the tip–surface interaction. The force-quadrature picture is such a scheme, which decouples information about the tip–surface interaction from the actual tip motion. We showed how the general idea behind approximative force reconstruction can be adapted to the force-quadrature picture, and we introduced an efficient way to extract the polynomial coefficients from the force quadratures.

We hope that in the future polynomial force reconstruction will be a useful method for many scientists and that new data-representation schemes will inspire innovative analysis methods.

## Experimental

The silicon oxide sample was cleaned in an oxygen plasma before measurements were perfomed in a Bruker Dimension 3100 AFM system. The cantilever (Bruker MPP-11120) was calibrated by a noninvasive thermal method [30] and had a resonance frequency of *f*_0_ = 229.802 kHz, a quality factor of *Q* = 396.9 and a spring constant of *k*_c_ = 16.0 N m^−1^. The slow surface approach velocity was 2 nm s^−1^.

PS (*M*_w_ = 280 kDa, Sigma-Aldrich) and PMMA (*M*_w_ = 120 kDa, Sigma-Aldrich) were spin-cast from toluene solution with a concentration of 0.53 %wt at a ratio of 3:1 (PMMA:PS). The sample was scanned in a Bruker Multimode 2 AFM system with a cantilever BS 300Al-G (Budget Sensors) having a resonance frequency *f*_0_ = 343.379 kHz, quality factor *Q* = 556.9 and spring constant *k*_c_ = 35.1 N m^−1^. The maximum free oscillation amplitude close to the surface was 30 nm and we scanned an image with 256 × 1024 pixels within 17 min.

For all measurements we used an intermodulation lock-in analyzer (IMP 2-32, Intermodulation Products AB) which synchronizes the signal generation and acquisition for measurement of the multifrequency response [31].

## Supporting Information

File 1 A movie showing the tip motion and the reconstructed tip–surface during an ImAFM approach measurement.
